# Supplementary material for: Environmental dynamics shape perceptual decision bias
Source: PLoS Comput Biol. 2023 Jun 8;19(6):e1011104. doi: 10.1371/journal.pcbi.1011104 (PMC10249884; doi:10.1371/journal.pcbi.1011104)
Supplement: S1 Text — (a) Low-contrast aligned bias (color) as a function of trials since the most recent context switch (columns) and the number of same context trials prior to the most recent context-switch (rows) for an assumed hazard rate of 10% and a low level of context cue reliability. (b) The pattern of aligned bias across a range of assumed levels of hazard rate and context cue reliability. Fig B. Comparison of goodness-of-fit of Dynamic GLM and Signal Detection Theory model. Low contrast data are shown as open symbols and high contrast data as filled symbols. Table A. AIC estimates for choice data collected during the test phase of the experiment under the Signal Detection Theory model and the Dynamic GLM. Table B. Average log likelihood for hold-out data predicted under four different variants of the Signal Detection Theory model (only high contrast trials included). Table C. Average log likelihood for hold-out data predicted under four different variants of the Signal Detection Theory model (only low contrast trials included). Table D. Each cell of the table reports the total log-likelihood of the held-out data for each subject. The rightmost column indicates the proportion of each subject’s total data the held-out fraction made up. (DOCX) [file pcbi.1011104.s001.docx]

**S1 Text**

**
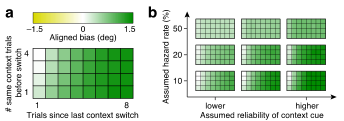
**

**Fig A.** Determinants of the Bayesian ideal observer’s aligned bias. (a) Low-contrast aligned bias (color) as a function of trials since the most recent context switch (columns) and the number of same context trials *prior* to the most recent context-switch (rows) for an assumed hazard rate of 10% and a low level of context cue reliability. (b) The pattern of aligned bias across a range of assumed levels of hazard rate and context cue reliability.

**Comparison of Signal Detection Theory model and Dynamic GLM**

We analyzed the choice data collected during the test phase of the experiment with two different models: a Signal Detection Theory based model (analyses in Figs 4 and 5), and a dynamic GLM (analysis in Fig 6). For each subject, the latter model provides a better description of the data. This is evident from the models’ AIC values, shown in Table A (the lower this value, the higher the quality of the model fit), and from a complementary analysis which compares each model’s normalized log-likelihood, split out for high and low contrast trials (Fig B).


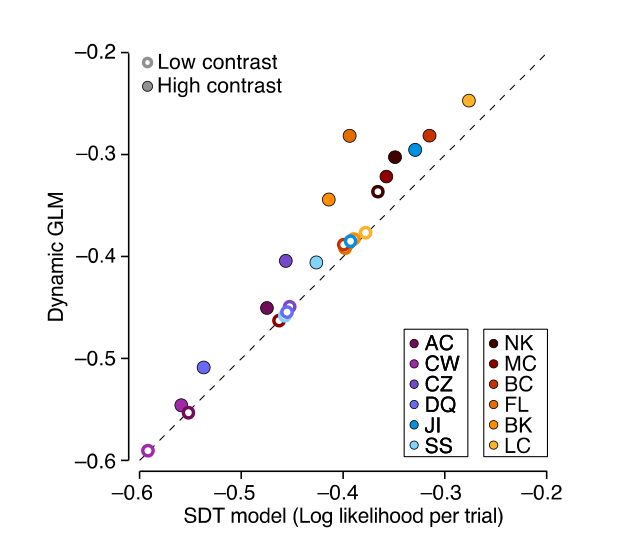


**Fig B.** Comparison of goodness-of-fit of Dynamic GLM and Signal Detection Theory model. Low contrast data are shown as open symbols and high contrast data as filled symbols.


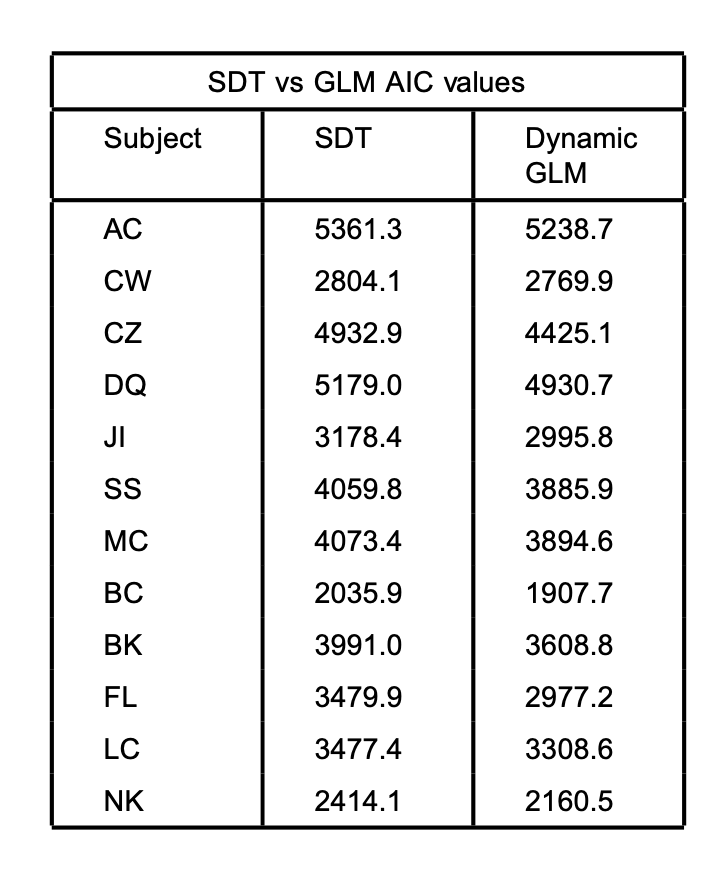


**Table A.** AIC estimates for choice data collected during the test phase of the experiment under the Signal Detection Theory model and the Dynamic GLM.

**Comparison of four Signal Detection Theory model variants**

We considered four variants of the Signal Detection Theory model. These variants differed in the number of free parameters used to describe the context-specific shift and spread of the orientation estimates. Model 1 was the most economically parameterized variant by imposing a symmetric context-specific bias for the non-uniform contexts (resulting in two free "shift" parameters) and a single context-independent level of estimation uncertainty (resulting in one free "spread" parameter). Model 2 differed from this variant by allowing for a context-specific level of estimation uncertainty (yielding three free "spread" parameters), while Model 3 instead allowed for asymmetric context-specific bias (yielding three free "shift" parameters). Finally, Model 4 was the least restrictive variant by allowing asymmetric context-specific bias (three free "shift" parameters) and a context-specific level of estimation uncertainty (three free "spread" parameters). To evaluate each model’s performance, we trained the models on all trials except for three pseudo-randomly chosen hold-out orientations (one per context). We then computed the quality of the models’ prediction for this hold-out set. We repeated this procedure 10,000 times. As can be seen in the bottom row of Tables B and C, Model 1 tended to perform best.


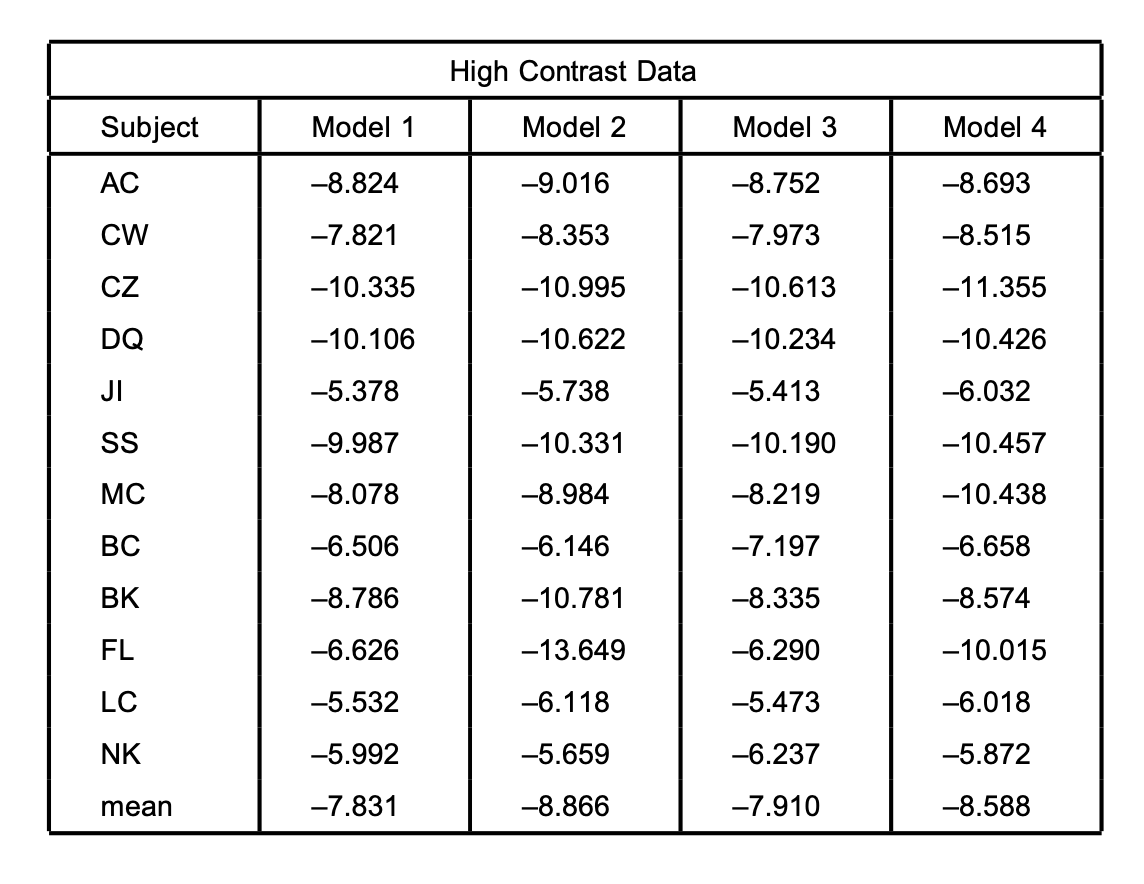


**Table B.** Average log likelihood for hold-out data predicted under four different variants of the Signal Detection Theory model (only high contrast trials included).
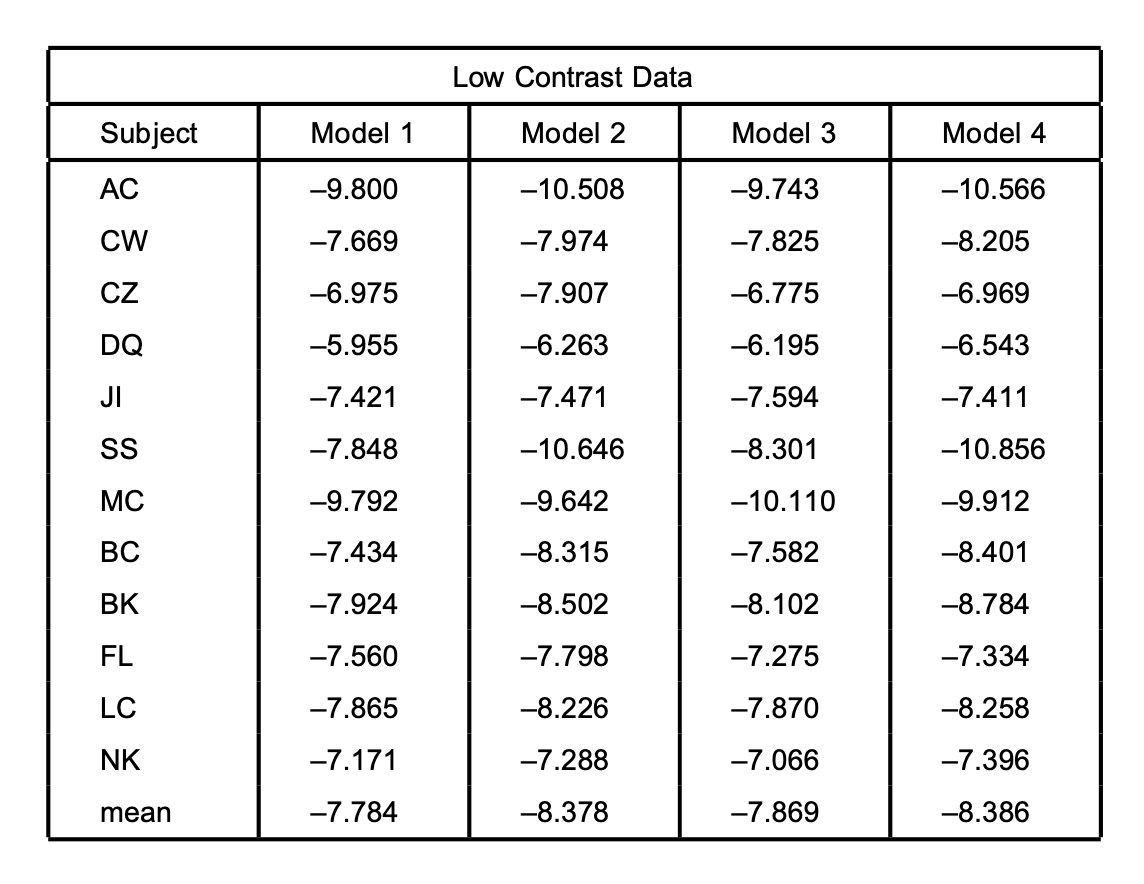


**Table C.** Average log likelihood for hold-out data predicted under four different variants of the Signal Detection Theory model (only low contrast trials included).

**Comparison of Dynamic and Static GLM**

We analyzed the choice data collected during the test phase of the experiment with a GLM that included a dynamic bias function (analysis in Fig 6). To assess the necessity of this model component, we conducted a cross-validation analysis in which we compared performance of this model with a variant that lacked this specific component (the "Static" GLM). These variants critically differ in the predictions they make for trials that occur after many trials within the same context. For this reason, we opted to use all trials that occurred between 1 and 4 trials after a context switch as training data and all trials that occurred 5 or more trials after a switch as hold-out test set. As can be seen in Table D, the dynamic GLM outperformed the static version in four of six veridical cue condition subjects and three of six ambiguous cue condition subjects.


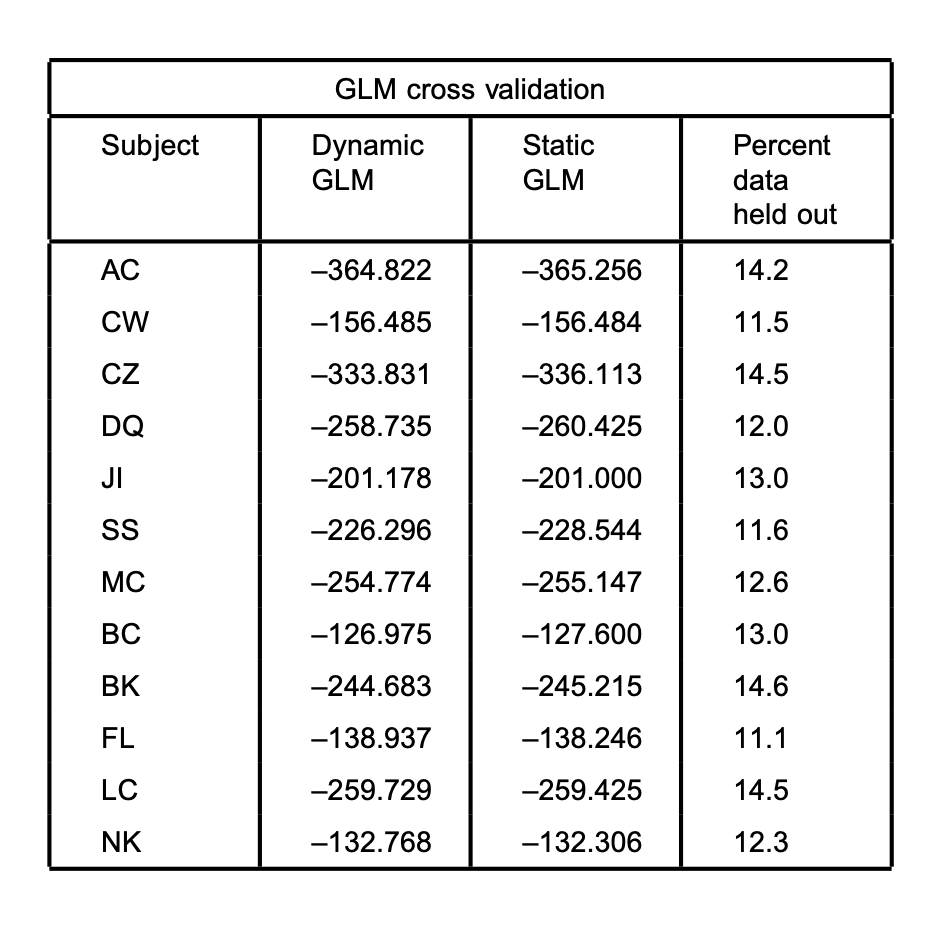


**Table D.** Each cell of the table reports the total log-likelihood of the held-out data for each subject. The rightmost column indicates the proportion of each subject’s total data the held-out fraction made up.
